# Microglia and Neuroinflammation: What Place for P2RY12?

**DOI:** 10.3390/ijms22041636

**Published:** 2021-02-06

**Authors:** Albert Gómez Morillas, Valérie C. Besson, Dominique Lerouet

**Affiliations:** UMR-S1144–Optimisation Thérapeutique en Neuropsychopharmacologie (OTeN), Faculté de Pharmacie de Paris, 4 Avenue de l’Observatoire, Université de Paris, 75006 Paris, France; albert.gomez.morillas1@gmail.com (A.G.M.); valerie.besson@u-paris.fr (V.C.B.)

**Keywords:** microglia, neuroinflammation, P2RY12, purinergic receptor

## Abstract

Microglia are immune brain cells involved in neuroinflammation. They express a lot of proteins on their surface such as receptors that can be activated by mediators released in the microglial environment. Among these receptors, purinergic receptor expression could be modified depending on the activation status of microglia. In this review, we focus on P2Y receptors and more specifically on P2RY12 that is involved in microglial motility and migration, the first step of neuroinflammation process. We describe the purinergic receptor families, P2RY12 structure, expression and physiological functions. The pharmacological and genetic tools for studying this receptor are detailed thereafter. Last but not least, we report the contribution of microglial P2RY12 to neuroinflammation in acute and chronic brain pathologies in order to better understand P2RY12 microglial role.

## 1. Introduction

Microglia are the main immune cells in the brain. These plastic cells display a variety of morphological and functional states in both healthy and pathologic conditions. Numerous studies demonstrated the involvement of microglial activation in different cerebral neuroinflammatory pathologies [1]. Microglia express a variety of cell-surface proteins that mediate their functions. Among these proteins, one can find some purinergic receptors. The latter are membrane-bound, ligand-gated ion channel (P2X) and G protein-coupled receptor (GPCR) (P2Y) for extracellular nucleotides involved in purinergic signaling. Glial cells release and respond to ATP and other purinergic molecules, released by injured cells or secreted, under both physiological and pathological conditions. Purinergic signaling plays an important role in regulating microglial activity. As microglia are involved in neuroinflammation, increasing studies have described the role of microglial purinergic receptors in brain inflammatory processes. In this review, we focus on P2Y receptors and more specifically on P2RY12. After a brief description of purinergic receptor families, we then review P2RY12 structure, expression and functions, especially the microglial one. We also summarize the pharmacological and genetic tools developed to study this purinergic receptor. Then, we highlight the contribution of microglial P2RY12 to neuroinflammation in nervous system pathologies.

## 2. P2RY12

### 2.1. Purinergic Receptor Families

Purinergic signaling and receptors were first described in the 1970s [2,3]. Purinergic signaling is involved in neurodevelopment and pathophysiological processes, such as cell proliferation, differentiation, neuron–glia cross-talking and inflammation [4].

The purinergic receptor family is divided into two subfamilies, P1 and P2 receptors, depending on their endogenous agonists [4,5]. On the one hand, the P1 receptor subfamily is constituted of four subtypes that are metabotropic receptors sensitive to adenosine. On the other hand, P2 receptors are activated by nucleoside di- and triphosphates (denosine diphosphate (ADP) and ATP; uridine di-(UDP) and triphosphate (UTP); UDP-glucose) and classified as P2X and P2Y receptors. The P2X receptor group is composed of seven subtypes that are ATP-gated ion channels permeable to cations (Na+, K+ and Ca2+). P2Y receptors are metabotropic receptors coupled with a G-protein that are activated by purines and pyrimidines [4,6]. P2RY12 is one of the eight members of P2Y receptor group expressed by humans [7]. Indeed, P2RY are divided into two subgroups based on ligand binding and the selectivity of G-protein binding. The first one consists of P2RY1, P2RY2, P2RY4, P2RY6 and P2RY11 that are coupled with Gq protein, which stimulates phospholipase C (PLC), resulting in calcium release from intracellular stores and protein kinase C (PKC) activation. One of these receptors, P2RY11 can also be coupled with Gs protein, which stimulates adenylate cyclase (AC) and increases the production of cyclic adenosine monophosphate (cAMP). On the contrary, the members of the second subgroup, P2RY12, P2RY13 and P2RY14, are coupled with Gi protein, resulting in a decreased cAMP production [6].

### 2.2. Structure

**Figure 1:**
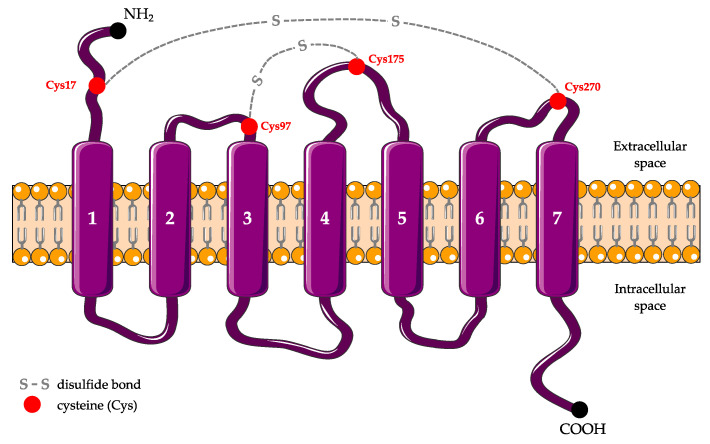
Schematic secondary structure of P2RY12. P2RY12 is composed of seven α-transmembrane domains connected by three extra- and intracellular loops. There are four extracellular cysteines (Cys) at positions 17, 97, 175, and 270 that form two disulfide bonds.

P2RY12 is a Gi-coupled receptor containing 342 amino acids and with a molecular weight of 39 kilodaltons. Its structure is composed of seven hydrophobic transmembrane regions of α-helice connected by three extra- and intracellular loops, and a carboxy-terminal helix VIII that is parallel to the membrane bilayer on the cytoplasmic side (Figure 1) [8]. All P2Y receptors possess four cysteine residues (Cys 17, 97, 175, 270) at their extracellular domain. Regarding P2RY12, these four cysteine residues form two disulfide bonds: the first one between the N-terminal domain and the third extracellular loop, and the second one between the first and the second extracellular loop. These disulfide bonds play a key role in the stimulation/inhibition of this receptor.

It is noteworthy that there is a marked difference between the agonist and the antagonist bound receptor structure. Indeed, when P2RY12 is activated, the receptor binding pocket is contracted, suggesting large-scale rearrangements in extracellular regions during the binding process. In this structure, both disulfide bonds are part of the agonist bound structure [6]. When it is antagonized, the receptor binding pocket is wide open and consists of two subdomains. In addition, the disulfide bond between the first and the second extracellular loop is missing.

### 2.3. Expression

Initially, P2RY12 was identified on platelets and in a lesser quantity in some regions of the brain. However, it is now well established that P2RY12 has a wider cell expression [9,10,11,12], as it is also expressed on vascular smooth muscle cells, brown adipocytes, cholangiocyte primary cilia, osteoblasts, osteoclasts, and several immune cells including dendritic cells and lymphocytes.

Within the brain, while there is no doubt that this receptor is expressed on the ramified processes of microglia [13,14,15], its expression in other brain cells is not so clear. Indeed, there are conflicting reports whether P2RY12 is expressed [9,12,16] or not on macrophages [13,15,17]. P2RY12 has also been detected on oligodendrocytes, suggesting that its expression might be a marker of demyelinating lesions in neuroinflammatory diseases, such as multiple sclerosis [17]. However, recently, Cserép and collaborators [18] have reported that P2RY12 expression is exclusively restricted to microglia, in agreement with previous studies [19,20,21].

The literature reports a stable P2RY12 expression during human brain development, including fetal phases [15]. In humans [22] and mice [23], P2RY12 expression is reduced in aged microglia compared to young microglia. Notably, human samples originated from patients with neuropathologies involving modification of microglial activation, which may have influenced the expression of P2RY12.

A sexually dysmorphic behavior in microglial P2RY12 expression was also observed in mice, as it was lower in four-month females than in males [23].

### 2.4. Functions

As mentioned above, P2RY12 is coupled with Gi-protein that inhibits AC and affects intracellular calcium concentration [4]. Consequently, its activation or inhibition affects many cellular and physiopathological responses.

#### 2.4.1. Platelet Aggregation

Platelet P2RY12 plays a crucial role in ADP-induced aggregation, which explains why this receptor is an established target of several antithrombotic drugs, such as clopidogrel or ticagrelor [24]. ADP-induced P2RY12 activation mediates AC inhibition through the activation of the Gαi2 G protein subtype, although effective coupling may also occur with Gαi1 and Gαi3 [25]. It is worth mentioning that ADP by itself is unable to cause the release of platelet dense granules, but its binding to both platelet receptors, P2RY1 and P2RY12, amplifies and sustains the secretion and aggregation induced by strong agonists, such as thrombin and thromboxane A2 [12]. Indeed, concomitant stimulation of P2RY1 and P2RY12 is necessary to generate normal ADP-induced platelet aggregation [6]. In response to ADP, P2RY1 triggers the mobilization of calcium from internal stores, which results in platelet shape modification and weak transient aggregation, while P2RY12 potentiating secretion and stabilizing aggregation. Therefore, P2RY12 is a key player in thrombus formation and stabilization.

Platelet activation also plays a fundamental role in inflammation, by modulating innate and adaptive immune responses. Indeed, upon ADP-induced P2RY12 activation, platelets release mediators from their granules, including various cytokines and chemokines, which can recruit and activate leukocytes, mainly neutrophils and monocytes [10,12,26]. Thus, the influence of activated platelets on inflammatory state has been demonstrated in several diseases, such as sepsis, rheumatoid arthritis, myocardial infarction and pulmonary inflammation.

#### 2.4.2. T Cell Activation

Among the other blood cells expressing P2RY12, it has been shown that the activation of this receptor on dendritic cells promotes specific T cell activation by increasing antigen endocytosis [27], while P2RY12 inhibition induces an immunosuppressive effect by decreasing antigen uptake [12].

#### 2.4.3. Vascular Effects

In vascular smooth muscle cells, P2RY12 plays an important role in the physiological functions of blood vessels, such as vasoconstriction, vasodilation and extracellular matrix production [12]. Moreover, ADP-induced P2RY12 activation seems to generate vascular inflammatory changes by upregulating monocyte chemoattractant protein-1 (MCP-1) and promoting monocyte adhesion [28]. MCP-1, one of the main mediators of vascular inflammation, triggers vessel wall inflammation by chemotactically inducing the monocyte migration into the vessel wall. Therefore, P2RY12 represents an important therapeutic target in atherosclerotic diseases.

#### 2.4.4. Bone Remodeling

There is also evidence that P2RY12 is involved in bone remodeling [11], as mice with a deficiency of this receptor present decreased osteoclast activity and show lower age-related bone loss.

#### 2.4.5. Microglia Functions: Motility and Migration

Microglia account for about 10% of brain cells and are the most abundant mononuclear phagocytes in the CNS. They participate in the maintenance of various homeostatic functions [29,30], but they are also the primary effectors of central inflammatory response to acute and chronic disorders [1,31]. Indeed, these cells display remarkable plasticity and are able to respond to a vast array of challenges. It is noteworthy that the microglial population is highly heterogeneous both in terms of cell density and transcriptional signature depending on brain regions, but also to age and gender, which underscores the numerous functions of microglia [30,32]. Despite these transcriptomic differences in brain regions, it has been demonstrated that all cells express a core profile of genes, among which is P2RY12. Moreover, it has been reported that in pathological conditions, such as neuroinflammation, microglia lose their transcriptomic homeostatic signature.

As the resident immune cells of the brain, it is well established that microglia survey the parenchymal environment by physically interacting with other cells such as neurons, other glial cells (oligodendrocytes, astrocytes) and cerebrovascular endothelial cells [33,34]. Their capacity to migrate is indispensable to tissue maintenance, but also in pathological conditions. Microglia exhibit two modes of motility [30,35]. Indeed, on the one hand, under physiological conditions, they continuously extend and retract their processes in all directions to survey the brain. On the other hand, under pathological conditions, activated microglia migrate toward the lesion site using chemoattractant gradient as a directional cue, in order to envelop sites of tissue damage with their processes. It is now established that these two motility modes differ mechanistically. Thus, although P2RY12 seems necessary to microglial directed motility (chemotaxis) in response to CNS injury [13], its role in the constant surveillance of the brain, as well as in the microglial ramification, appears to be mainly dependent on the tonic activity of the newly described two-pore domain K+ channel, THIK-1 (TWIK-related halothane-inhibited K+ channel) [35]. Thus, THIK-1 maintains the “resting” potential of microglia. In line with these data, Sipe and collaborators [36] also reported that P2RY12 signaling contributed to but was not necessary to maintain microglial ramified morphology.

**Figure 2:**
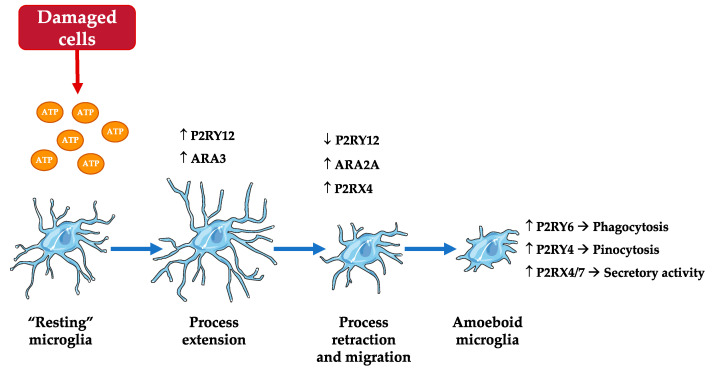
Purinergic receptors and microglial activation states (modified from [37]). In response to ATP release from damaged cells, P2RY12 stimulation triggers the extension of microglial processes towards the site of injury, in cooperation with the adenosine receptor (AR) A3. Both these receptors are upregulated during this step. Then, P2RY12 downregulation and ARA2A upregulation induce the retractation of these processes. Microglial migratory activity also depends on the interaction between P2RY12 and P2RX4. After total retraction of processes, microglia adopt an amoeboid morphology, and exert phagocytosis, pinocytosis or secretory activity depending on the receptor involved. ↑: upregulation or increased activation; ↓: downregulation or reduced activation.

In healthy brain, “resting” microglia exhibit a ramified shape, but in pathological brain, they progressively adopt an amoeboid shape once activated. It is noteworthy that the purinergic receptor density is modified depending on microglial activation state [37] (Figure 2). Indeed, in response to the chemoattractant or “find-me” signal, ATP, released from damaged cells [38,39], the first step of microglial activation is the stimulation of P2RY12. The latter triggers the extension of microglial processes towards the site of injury, in cooperation with another microglial receptor, the adenosine receptor (AR) A3. Then, these processes retract due to P2RY12 downregulation and ARA2A upregulation. Microglial migratory activity towards the source of released ATP also depends on the interaction between P2RY12 and P2RX4. After total retraction of microglial processes, microglia adopt an amoeboid morphology and exert phagocytosis (P2RY6), pinocytosis (P2RY4) or secretory activity (P2RX4 and 7) depending on the purinergic receptor involved.

This microglial chemotaxis via P2RY12 is necessary for the clearing of infected cells or cellular debris, and for tissue repair. Furthermore, Lou and collaborators [21] reported that following blood–brain barrier (BBB) breakdown, microglial chemotaxis via P2RY12 induces the rapid closure of BBB by forming a dense aggregate at the site of injury.

**Figure 3:**
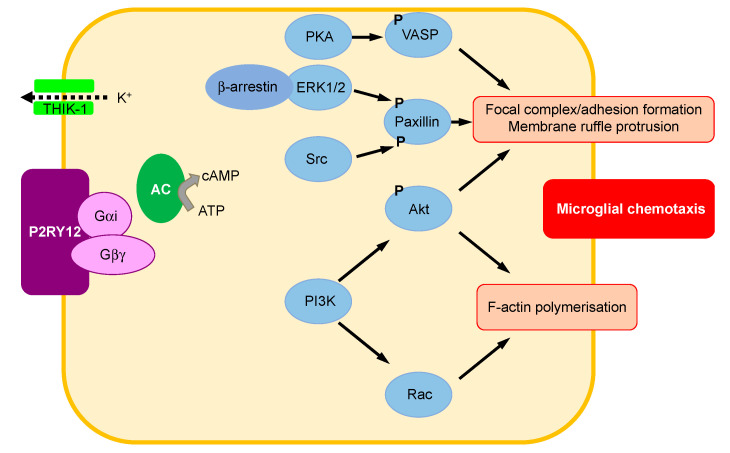
Major signal pathways involved in microglial motility and migration after P2RY12 activation. (1) Once released from Gαi, Gβγ activates adenylate cyclase (AC), which induces cyclic adenosine monophosphate (cAMP) increase, phosphorylation of vasodilator-stimulated phosphoprotein (VASP) by protein kinase A (PKA), and then membrane ruffle formation and chemotaxis via the regulation of focal adhesion formation/maturation. (2) The recruitment of β-arrestin recruits and activates extracellular signal-regulated kinase (ERK) 1/2, inducing paxillin phosphorylation which is necessary for adhesion disassembly during chemotaxis. (3) The activation of Src through Gαi triggers the phosphorylation of paxillin, which is essential to focal adhesion assembly. (4) Gαi and Gβγ activate phosphoinositide 3-kinase (PI3K), promoting Akt (=protein kinase B) and Rac activation that are both involved in F-actin polymerization. (5) P2RY12 activation potentiates the activity of TWIK-related halothane-inhibited K+ channel (THIK-1) involved in microglial ramification regulation and surveillance of healthy brain.

To go into further detail, several signaling pathways have been reported to be involved in microglial chemotaxis (Figure 3) [30,40].

When released from Gαi, Gβγ can also transiently activate AC, which subsequently induces cAMP increase, phosphorylation of vasodilator-stimulated phosphoprotein (VASP) by protein kinase A (PKA), resulting in membrane ruffle formation and chemotaxis via the regulation of focal adhesion formation/maturation. However, prolonged phosphorylation of VASP perturbs this mechanism, resulting in defective chemotaxis. Therefore, balanced regulation of phosphorylation and dephosphorylation of VASP is necessary for efficient chemotaxis.

In addition, on the one hand, P2RY12 stimulation triggers the recruitment of β-arrestin, which in turn recruits and activates extracellular signal-regulated kinase (ERK) 1/2, inducing paxillin phosphorylation at Ser83 that is required for adhesion disassembly during chemotaxis. On the other hand, the activation of Src through Gαi triggers the phosphorylation of paxillin at Tyr31, which is essential to focal adhesion assembly.

Thus, P2RY12 stimulation induces the activation of phosphoinositide 3-kinase (PI3K) α and γ via Gαi and Gβγ, respectively, which activates Akt (=protein kinase B) and Rac. Data suggest the existence of a positive feedback loop between PI3K and F-actin polymerization regulated by Rac GTPase [41]. Ras can further activate PI3K, increasing the F-actin polymerization. This signaling cascade plays an essential role in the regulation of cell polarity, which is useful to sense and respond to environmental concentration gradients. The activation of P2RY12 was also reported to potentiate the activity of THIK-1, which regulates microglial ramification and surveillance of the brain in healthy conditions [35].

In recent years, a novel form of microglia–neuron interaction called microglial process convergence (MPC) has been described [42]. The proposed model suggests that an excessive glutamate release activates neuronal N-methyl-D-aspartate receptors, which triggers the release of chemokine fractalkine, CX3CL1 (C-X3-C motif chemokine ligand), from neurons and consequently the activation of microglial CX3CR1 (C-X3-C motif chemokine receptor 1). Then, CX3CR1 activation induces microglial IL-1β release that stimulates neuronal dendrites and subsequently triggers the release of ATP. Last, the latter elicits the localized convergence of microglial processes through P2RY12.

It has also become clear that microglia are far more active in the healthy brain than previously thought [43]. Although a dynamic microglia–neuron crosstalk has already been observed at synaptic structures [37,44], a recent publication identified the site of interaction between microglia and neurons at the cell body rather than at synaptic elements in both mice and humans [18]. While the activation of P2RY12 was previously mainly associated with pathological conditions, the authors also highlight the importance of these receptors under physiological conditions. Therefore, microglia continuously monitor neuronal status through somatic junctions, rapidly responding to neuronal changes and initiating neuroprotective actions.

The importance of P2RY12 signaling in microglia–neuron interactions was also highlighted during plasticity in healthy conditions [36].

As mentioned above, purinergic receptor density varies depending on microglial activation state. Thus, data suggest that P2RY12 is a useful marker for the identification of healthy microglia and to discriminate activated microglia from quiescent microglia [15], as its expression is downregulated during the inflammatory phenotype shift [13]. Moreover, “resting” microglia show no or few P2RY6, but a high level of P2RY12. When activated, the expression of P2RY6 is increased while that of P2RY12 is decreased, suggesting a functional modal shift from chemotaxis to phagocytic function [45]. 

Interestingly, the microglial P2RY12 response to ATP appears to be age-dependent [46]. Thus, once P2RY12 is activated, aged microglia become less dynamic and ramified, while the opposite occurs in young microglia.

In addition, microglial P2RY12 expression is phenotype-dependent. Indeed, P2RY12 is highly expressed in quiescent and activated non-inflammatory M2 microglia [36,38], but less expressed in activated M1 microglia [14], suggesting a role of this receptor in microglial polarization.

## 3. Pharmacological and Genetic Tools for Studying P2RY12

As mentioned above, P2RY family is activated by purines and pyrimidines [4,6]. For P2RY12, ADP is the native agonist, while ATP and ATP nucleotides and dianedine nucleotides can act as partial agonists or antagonists of the receptor [6]. ATP is a ubiquitous intracellular molecule, released by injured cells or in response to physiological brain activity, which plays an important role as a danger signal in the extracellular space [47]. Structurally, ATP differs from ADP in having a γ-phosphate group that is crucial for the antagonism of the receptor [48]. Interestingly, the cluster differentiation 39 (CD39) regulates P2 receptor-mediated functions by converting extracellular ATP to ADP, and then to AMP [49,50]. In the brain, CD39 is exclusively expressed on microglia, and on endothelial and smooth muscle cells of the vasculature [51]. Therefore, it is involved in nucleotide signaling regulation between neurons or astrocytes associated with microglial ramifications, and also in the regulation of blood flow and thrombogenesis. Other molecules, such as farnesyl pyrophosphate, an intermediate in cholesterol biosynthesis structurally related to ADP, have also been shown to act as endogenous, low affinity antagonists [52].

Over the years, many synthetic ligands have been developed to improve knowledge on P2RY12 pharmacology. Thus, some potent molecules, such as 2-methylthio-ADP and 2-methylthio-ATP, which present a high affinity for P2RY12, have been discovered. Both these potent agonists are close analogues of ADP and ATP, respectively [6,7].

Currently, several P2RY12 antagonists are used in pharmacotherapy to reduce platelet aggregation. Consequently, they are highly effective to prevent and treat cardiovascular events, such as myocardial infarction or stroke [11]. These antiaggregant ligands are divided into two drug classes: thienopyridines and nucleoside–nucleotide derivatives (Table 1) [12]. The thienopyridine compounds clopidogrel, prasugrel, and ticlopidine are prodrugs that need to be enzymatically converted by the hepatic cytochrome P450 into active metabolites, and consequently present a delayed onset of action [53]. Their active metabolites covalently bind to P2RY12 by forming a disulfide bond with the cysteine residues, resulting in an irreversible inhibition of the receptor [54]. By contrast, the nucleoside–nucleotide derivatives, such as cangrelor and ticagrelor, do not require hepatic metabolism and are part of a new generation of reversible direct-acting compounds. They are faster, more potent and more predictable than thienopyridines [12]. They promote a rapid inhibition of P2RY12 through a direct and reversible binding [53]. Therefore, both these antagonists are widely prescribed as antiplatelet treatment.

Recently, new analogues of P(1), P(4)-di(adenosine-5’) tetraphosphate (Ap4A) and UTP have proved to have an anti-aggregant effect. While adenosine analogues simultaneously antagonize P2RY1 and P2RY12 [63], those of UTP exclusively inhibit P2RY12 [64].

Suramin and reactive blue-2 block also act as P2RY12 antagonists [6], but at high micromolar concentrations (30–100 µM), the first one also blocks all nucleotide-sensitive P2RY except P2RY4, and the second one blocks P2RY1 and P2RY6 [65]. PSB-0739, an analogue of reactive blue-2, is a potent and competitive antagonist of P2RY12. 

The other novel compounds that have demonstrated an inhibitory effect on P2RY12 are 6-amino-2-thio-3H-pyrimidin-4-one derivatives, morpholine analogues, piperazinyl glutamates, derived phosphonates, salvianolic acids, flavonolignans, ethyl 6-aminonicotinate acyl sulfonamides and related sulfonamide derivatives [6,11].

Beside pharmacological tools, the genetic approach is very useful to understand the functions of a protein by changing its expression in specific conditions. To study P2RY12, two approaches have been developed: (1) the knockout (KO) model that makes the gene of interest inoperative, and (2) the silent ribonucleic acid (siRNA), in which a small RNA interferes with the expression of specific genes with complementary nucleotide sequences by degrading mRNA after transcription, preventing translation. It should be noted that P2RY12-deficient mouse strains present decreased platelet aggregation and increased bleeding time [66].

## 4. Contribution of Microglial P2RY12 to Neuroinflammation

Neuroinflammation is defined as an inflammatory response within the brain or spinal cord that is triggered by infection or injury. It is mediated by the production of cytokines, chemokines, reactive oxygen species and secondary messengers by resident CNS cells and peripherally infiltrating immune cells [67,68,69]. Neuroinflammation is recognized as a hallmark of neurological disorders, as excessive and uncontrolled neuroinflammation induced injury and neural death. However, there are several degrees of neuroinflammatory responses, some of which are beneficial for the repair of the injured CNS. Microglia, which play key roles in mediating these neuroinflammatory responses, perfectly reflect this “friend-foe facet”, which is linked to their polarization states.

As mentioned above, in their “resting” state microglia constantly explore the local environment through their multiple branches and highly motile processes during normal physiological conditions. These processes are in continuous motion, protruding and retracting to cover long distances and survey large brain areas. In the case of brain injury or neuroinflammation, microglia become highly activated and gradually change from a ramified shape to an amoeboid form, which is associated with phagocytosis and proinflammatory function. This ability to rapidly react has been described as “microglial activation”, which will depend on the type of stimulus, time after stimulation and factors present in the local environment. Thus, once activated, microglia can adopt heterogeneous phenotypes ranging from the “classical” proinflammatory phenotype (M1-like) to the “alternative” anti-inflammatory one (M2-like) depending on the stimulus and their CNS microenvironment [29]. The M1-like microglia are typically induced by exposure to bacterial products such as lipopolysaccharide, or proinflammatory cytokines, such as interleukin (IL)-1β, interferon γ and tumor necrosis factor α, and trigger the production of high levels of proinflammatory cytokines and cytotoxic oxidative metabolites. Ultimately, M1-like microglia induce inflammation and neurotoxicity. Regarding the M2-like microglia, three distinct subsets have been described [33,70]: M2a, involved in repair and regeneration; M2b, an immune-regulatory phenotype; M2c, an acquired-deactivating phenotype. The M2a phenotype is induced by anti-inflammatory cytokines, such as IL-4 and IL-13, and contributes to tissue remodeling. In response to specific anti-inflammatory factors, such as IL-10, glucocorticoids and growth factors, microglia adopt a M2c state, involved in inflammation resolution and tissue remodeling. Last, the M2b phenotype, induced by exposure to immune complexes and ligands of toll-like receptors, seems to have an immunoregulatory effect. M2-like microglia are also characterized by an elongated shape and a higher level of F-actin than M1-like microglia. Nevertheless, it is currently recognized that microglia display a wide range of reaction states that are far more complex than this M1/M2 classification [71]. Indeed, Stratoulias and collaborators [72] have recently proposed that microglia might form a community of cells in which each member or subtype displays distinct properties, performs unique physiological functions, depending on their regional distribution, gene and protein expression, and responds differently to stimuli.

Relevant to their role as immune sentinels, microglia express a wide range of receptors, among which is the purinergic one [29]. The purinergic system is one of the fundamental signaling systems that establish microglial behavior in a wide spectrum condition [47]. Moreover, this system controls inflammatory responses in complex ways [73]. While the main functions of inflammation are to limit tissue damage and promote tissue repair, inappropriate inflammatory responses, particularly when chronic, may lead to toxicity and cell death. Among P2 receptors, P2X4 and 7 and P2Y6, 12 and 13, pertinently expressed on microglia [47], we focused on the role of P2RY12 in several neuroinflammatory diseases. Indeed, as first reported by Haynes and collaborators [13], P2RY12 is a primary site through which nucleotides mediate rapid microglial responses to brain injury. These authors were the first to report a robust expression of P2RY12 in resting microglia and a diminution after its morphological transition and activation. Some years later, Amadio and collaborators [17] highlighted the gradual loss of P2RY12 immunoreactivity in mice, rats and humans as an early marker of neuroinflammation and microglial activation. Since then, it has been demonstrated that P2RY12 expression level changes depending on microglial phenotype. Indeed, P2RY12 is highly expressed on quiescent and activated non-inflammatory M2 microglia [36,38] and less expressed in activated M1 microglia [14]. Consequently, loss of P2RY12 expression in microglia has been reported in most of the neuropathologies related to neuroinflammation [15].

The neuroinflammatory cascade relies on the activation of cytosolic multiprotein complexes called “inflammasomes” [74]. The nod-like receptor protein 3 (NLRP3), which is the most investigated one, is notably present on microglia. The aberrant activation of this inflammasome signaling has been demonstrated to contribute to the development of several neurological diseases, such as cerebral ischemia, traumatic brain injury (TBI), Alzheimer’s disease (AD) and multiple sclerosis (MS) [75]. Notably, recent data suggest that microglial P2RY12 could be involved in NLRP3 inflammasome activation [76], which strengthens the role of P2RY12 in inflammation.

We propose to present herein the studies that were conducted, using genetic or pharmacological tools for P2RY12, in order to understand the role of P2RY12 in acute and chronic cerebral diseases. However, targeting P2RY12 with antagonists in brain diseases only makes sense under the assumption that ATP (required to activate P2RY12) is a danger signal in brain diseases [77].

### 4.1. Cerebral Ischemia

Webster and collaborators [78] were the first to report the deleterious role of P2RY12 in cerebral ischemia. Indeed, in vitro, the addition of P2RY12 deficient microglia (obtained by using siRNA) to neuron–astrocyte cultures reduced neurotoxicity following oxygen–glucose deprivation (OGD). Moreover, the migration was less significant in microglia from P2Y12-/- mice than in those of wild-type (WT) in response to OGD-conditioned neuronal media. In addition, P2RY12+/- mice or clopidogrel-treated mice subjected to global cerebral ischemia presented less neuronal injury than control mice. It is worth noting that homozygote P2RY12-/- ischemic mice could not be used in this study as they suffered high mortality and showed highly variable neuronal damage. Moreover, in a permanent model of focal ischemia in rats, blockade of microglial P2RY12 with ticagrelor reduced the evolution of ischemic lesion and the associated neurologic impairment [79]. This effect was associated with the inhibition of P2RY12-mediated microglial activation and chemotaxis as, in primary culture of microglia, ticagrelor and cangrelor totally inhibited ADP-induced chemotaxis. Lastly, the authors also observed a spatial gradient in microglial P2Y12 expression, reflecting microglial activation status. Thus, P2RY12 expression was downregulated in the core of the lesion compared to the penumbra area.

### 4.2. Traumatic Brain Injury

According to recent studies, neuroinflammation is a key player in chronic neurodegeneration and related neurological dysfunction following TBI. The activation of microglia and macrophages occurs in association with tissue damage and late cognitive disorder after TBI [80]. TBI induces the release of microglial-derived microparticles expressing P2RY12 in the systemic circulation [81]. The cortical injection of these secreted microparticles, loaded with pro-inflammatory molecules, induces neuroinflammation in non-injured mice and also contributes to spreading the neuroinflammatory response at more distant sites. However, to date, the role of P2RY12 remains to be established in TBI.

### 4.3. Epilepsy

Recent evidence also implicates glial cells, specifically microglia, and neuroinflammation in the pathogenesis of epilepsy. Nowadays, the most widely used animal seizure models include chemically-induced models using kainic acid or pilocarpine [82]. An increased number of microglia primary processes was observed in the hippocampus during kainic acid-induced seizure activity [83,84]. Furthermore, P2RY12 genetic deletion in mice exacerbated seizure outcome associated with reduced microglial processes [84], suggesting a neuroprotective role of P2RY12-dependent microglial process extension in epilepsy.

Whatever the brain pathology, it is well known that microglia participate in the clearance of dead cells or debris through the release or leakage of ATP by injured cells into extracellular space. As described above, released ATP is detected through P2RY12 and triggers microglial rapid migration and process extension to the lesion site. However, UDP/UTP is leaked in hippocampal neuron following kainic acid administration in vivo and in vitro, and those nucleotides can specifically activate P2RY6. P2RY6 activation by UDP triggers phagocytosis in a concentration-dependent manner. Interestingly, UDP does not efficiently activate P2RY12, nor can ATP/ADP act on P2RY6. Inoue [85] reported an increment in mRNA for P2RY6 in activated microglia in hippocampal CA1 and CA3 regions, where neuronal cell death appeared following kainic acid administration in rats.

### 4.4. Glial Tumors

Recent studies allow knowledge of a little more about the role of microglia in the development of glial tumors. Indeed, P2RY12 expression was analyzed in human astrocytoma of various malignancy grades in relation to M1 and M2 microglial phenotype activation profiles [86]. P2RY12 mRNA levels and P2RY12 membrane-bound localization were inversely correlated with increasing malignancy grade. Interestingly, low-grade gliomas expressed P2RY12 in cytoplasm, while, in high-grade tumors, P2RY12 expression shifted to the nucleus. On the one hand, cytoplasmic P2RY12 expression was associated with the expression of M1-proinflammatory microglial markers. On the other hand, nuclear P2RY12 expression was associated with the expression of M2-anti-inflammatory markers. Thus, microglial P2RY12 expression and localization are directly related to tumor grade and predominant microglial phenotype. Moreover, the expression of P2RY12 was also positively correlated with overall survival times.

### 4.5. Alzheimer’s Disease

Neuroinflammation is considered as a key pathological process in neurodegenerative diseases of aging, including AD [87]. The latter is characterized by a progressive extracellular amyloid beta (Aβ) plaque formation and phosphorylated tau aggregation, triggering a synaptic and neuronal cell loss. Sánchez-Mejías and collaborators [88] demonstrated that in vitro soluble phospho-tau from AD hippocampus were toxic for murine microglial cells. Microglial P2RY12 expression is reduced in human AD hippocampus [88], while no change was observed in parenchymal, non-plaque associated microglia in human AD and in the mouse amyloid model of AD, APPPS1 [89]. In line with these data, in the hippocampus of AD patients, the majority of microglia around Aβ plaques showed no expression of P2RY12, while microglia at a distance from the plaque core expressed P2RY12 [15]. Recently, Walker and collaborators [87] have observed that microglial P2RY12 expression differed depending on the type of plaques or tangles they were associated with. Thus, authors proposed that the closer to Aβ plaques microglia were, the lower P2RY12 expression was, suggesting that low-negative P2RY12 microglia is a marker of inflammatory area within Aβ plaques. Nevertheless, recent gene expression profiling studies of microglia isolated from AD tissues, from humans and animals, have provided large amounts of data on microglial properties and identified potentially new phenotypic markers for studying microglia in diseases.

### 4.6. Multiple Sclerosis

Some diseases or lesions produce damage in myelin, resulting in a process called demyelination. Chronic neuroinflammatory diseases, such as MS, are characterized by the appearance of demyelinating plaques. It is a heterogenous disease characterized by a leukocyte infiltration in the CNS, a demyelination of grey and white matter, and consequently, an axonal loss [90]. Microglia/macrophages, which accumulate at the sites of active demyelination and neurodegeneration in MS brain, are major players in the disease process [91]. In MS, extracellular ATP is an important mediator of CNS system pathology that can cause oligodendrocyte excitotoxicity [92]. An analysis of the cellular distribution of P2RY12 protein, performed in post-mortem cortex samples from MS patients as well as healthy human subjects, showed that P2RY12 was present in myelin and interlaminar astrocytes but absent from protoplasmic astrocytes in deeper cortical layers, from microglia/macrophages, and from intact demyelinated axons [92]. More recently, an increase in microglial activation has been observed in the normal-appearing white matter of MS patients in comparison to controls, associated with a reduction in P2RY12 expression [91]. Moreover, P2RY12 was totally absent in active and slowly expanding lesions. Interestingly, inactive lesions contained very few microglia but these cells expressed P2RY12, suggesting that the loss of P2RY12 immunoreactivity was associated with the lesion activity, i.e., enhanced inflammation, which was confirmed in other studies [15,90,93]. Similar results were reported in the experimental cuprizone demyelination model [94] and in the experimental autoimmune encephalomyelitis (EAE) model [93]. However, a target that disappears in the active phase of brain disease, such as P2RY12, presents actually low therapeutic interest.

### 4.7. Amyotrophic Lateral Sclerosis

Although the mechanisms are not well understood, there is evidence that the immune system plays a role in the pathogenesis of amyotrophic lateral sclerosis (ALS). Indeed, in both ALS patients and animal models, inflammatory responses were reported. Moreover, microglia and astrocytes, which are activated during disease progression, could contribute to neuronal death. Using the superoxide dismutase 1 (SOD1) mouse model of ALS, Butovsky and collaborators [95] reported that P2RY12 gene expression was decreased during disease progression and that protein expression was even absent in spinal cord microglia. Similar gene downregulation was also found in spinal cord from ALS patients.

## 5. Conclusions

In this review, we have attempted to give an overview of the importance of microglial P2RY12 in neuroinflammation. Initially, P2RY12 expression was used as a marker of non-activated/homeostatic microglia, the key cell of neuroinflammation, and a decreased expression was associated with proinflammatory activated microglia. However, modification of P2RY12 expression seems to be different depending on neuroinflammatory pathologies and on the microglial populations associated with active inflammatory areas. Even though investigations need to be conducted to clarify this point, an increasing number of studies have demonstrated its role in inflammatory brain diseases, highlighting its potential as a therapeutic target. However, given the prominent role of peripheral P2RY12, the therapeutic potential of P2RY12 antagonists in brain-related diseases should always be considered in conjunction with the potential immune and hemorheological risks. Moreover, immune and hemorheological effects should also be considered as a potential mechanism of action of the systemic administration of P2RY12 antagonists in the outcome of brain diseases. In addition, it is well described that the hemorheological effect of P2RY12 antagonist is observed in a predominant subset of individuals, whereas a minority of patients may draw no benefit or even experience detrimental effects. Thus, one may wonder whether similar interindividual variability of the role of P2RY12 in microglia could exist.

## Figures and Tables

**Table 1:** P2RY12 antagonists.

	Antagonist	IC_50_ (µM)	Binding	References
**Natural antagonist**	ATP	0.0003	Reversible	[48,55]
**Thienopiridines**	2-oxo-clopidogrel (clopidogrel metabolite)	0.1	Irreversible	[6,56]
	R-138727 ^1^ (prasugrel metabolite)	1		[57]
	UR-4501 ^2^ (ticlopidine metabolite)	2		[58,59,60]
**Nucleotide-Nucleoside derivatives**	Ticagrelor	0.003	Reversible	[6]
	Cangrelor	0.0008		[6]
**Other molecules**	Suramin	3	Reversible	[6,61]
	Reactive blue-2	0.025		[6]
	PSB-0739	0.0002		[6]
	AZD1283	0.01		[62]

^1^ (2Z)-2-[1-[2-cyclopropyl-1-(2-fluorophenyl)-2-oxoethyl]-4-sulfanylpiperidin-3-ylidene]acetic acid; ^2^ ([1-[(2-chlorophenyl)methyl]-4-mercapto-3-piperidinylidene]acetic acid.

## Abbreviations

Aβ	Amyloid beta
AC	Adenylate cyclase
AD	Alzheimer’s disease
ADP	Adenosine diphosphate
ALS	Amyotrophic lateral sclerosis
AR	Adenosine receptor
ATP	Adenosine triphosphate
BBB	Blood-brain barrier
cAMP	Cyclic adenosine monophosphate
CD39	Cluster differentiation 39
CX3CL1	C-X3-C motif chemokine ligand
CX3CR1	C-X3-C motif chemokine receptor 1
Cys	Cysteine
EAE	Experimental autoimmune encephalomyelitis
ERK	Extracellular signal-regulated kinase
GPCR	G protein-coupled receptor
IL	Interleukin
KO	Knockout
OGD	Oxygen-glucose deprivation
MCP-1	Monocyte chemoattractant protein-1
MPC	Microglial process convergence
mRNA	Messenger ribonucleic acid
MS	Multiple sclerosis
NLRP3	Nod-like receptor protein 3
PI3K	Phosphoinositide 3-kinase
PKA	Protein kinase A
PKC	Protein kinase C
PLC	Phospholipase C
siRNA	Silent ribonucleic acid
SOD1	Superoxide dismutase 1
TBI	Traumatic brain injury
THIK-1	TWIK-related halothane-inhibited K+ channel
UDP	Uridine diphosphate
UTP	Uridine triphosphate
VASP	Vasodilator-stimulated phosphoprotein
WT	Wild-type
